# Morphogenetic Metals through Topology‐Driven Stiffness Changes and Electrochemical Activation

**DOI:** 10.1002/smll.202510823

**Published:** 2025-10-31

**Authors:** Jungtaek Kim, Yash Agrawal, Zakaria Hsain, James K. Guest, James H. Pikul

**Affiliations:** ^1^ Department of Mechanical Engineering University of Wisconsin‐Madison Madison WI 53706 USA; ^2^ Department of Civil and Systems Engineering Johns Hopkins University Baltimore MD 21218 USA; ^3^ Department of Mechanical Engineering and Applied Mechanics University of Pennsylvania Philadelphia PA 19104 USA; ^4^ Present address: Hydrogen and Fuel Cell Technologies Office U.S. Department of Energy Washington, DC 20024 USA

**Keywords:** auxetic, electrochemical activations, morphogenetic metals, stiffness changes, topology‐driven

## Abstract

Morphogenesis enables the adaptive capabilities of living organisms and has been realized in soft materials that heal, change stiffness, and grow. Metals, despite their importance, do not have the tools to enable morphogenesis near ambient temperatures because metal atoms require intense temperatures and energy to move. This paper reports a method for reversibly controlling the physical properties of metal lattices with minimal energy, power, and material input through electrochemically activated topology changes. Switching between two topological states in individual cells enables programmable material moduli between 1.1 MPa and 2.6 GPa. The room temperature electrochemical morphogenesis is reversible and provides in situ phase control of a vibrating mass. A topology optimization technique that solves for two extreme topologies with minimal change in part volume realizes metamaterials that switch between negative and positive Poisson's ratios. The presented approaches for changing mechanical properties through topology provide a new path for endowing metals with the adaptive characteristics of organisms.

## Graphical abstract

Morphogenesis in metals is achieved through reversible, electrochemically driven topological changes, which switch stainless‐steel lattices between compliant and stiff states (1.1 MPa–2.6 GPa) at room temperature and with minimal energy input. These topological transitions also allow programmable vibration control and auxetic behavior, establishing a pathway toward metallic materials that adapt their mechanical properties like living organisms.

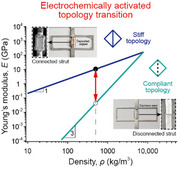

## 1 Introduction

The ability of a cell or tissue to change its shape through morphogenesis enables the proliferation of life by providing an organism with control over itself and its environment.^[^ ^1^, ^2^, ^3^ ^]^ Humans have sought to mimic morphogenesis by making materials^[^ ^4^, ^5^, ^6^ ^]^ that heal,^[^ ^7^ ^]^ change stiffness,^[^ ^8^ ^]^ and have controlled growth.^[^ ^9^, ^10^ ^]^ The mechanistic insights into these processes have realized materials that improve robotics, aviation, and medical devices^[^ ^11^ ^]^ and are enabling abilities impossible to achieve with conventional static materials.^[^ ^12^, ^13^, ^14^ ^]^ Despite these advances, morphogenesis has been largely limited to soft materials that are highly mobile or compliant at room temperature.^[^ ^15^ ^]^

Structural metals are incredibly strong, stiff, and tough materials that have paved the foundation of civilization,^[^ ^16^ ^]^ but they are not designed to change their shape or properties during use, as this requires high temperatures or pressures and, often, a transition to a molten phase that cannot retain their form and solid properties. These challenging processing conditions are also the reason structural metals are not found in biology. Although there is no direct bio‐inspiration for metal morphogenesis, structural metals have still benefited from bio‐inspired design. Most notably in cellular metals that improve their specific or volumetric properties by adding porosity.^[^ ^17^, ^18^ ^]^ The pore topology and relative density of cellular metals dictate their mechanical properties.^[^ ^19^ ^]^ Adding more pores, or reducing relative density can smoothly reduce the stiffness and strength of cellular metals, but the degree to which this change occurs depends on the pore topology. Materials with the same relative density but distinct topologies can have several orders of magnitude difference in their mechanical properties^[^ ^20^, ^21^ ^]^ due to, for example, changes from bending‐dominated to stretching‐dominated behavior classified by Maxwell's criteria.^[^ ^22^ ^]^ There are many models that can predict cellular stiffness from topology, or design lattices to achieve optimal properties at a given density.^[^ ^20^, ^22^, ^23^, ^24^, ^25^, ^26^, ^27^ ^]^ However, once fabricated, the material properties are unchanged, except for degrading over time. Recent research has attempted to remove this limitation by enabling healing‐like properties or adjusting stiffness through phase changes,^[^ ^28^, ^29^, ^30^ ^]^ but the programmable range of stiffness changes is limited by the soft, low‐melting temperature metals used, and large energy inputs are needed to change the metal temperature. Metal morphogenesis could be as transformative as soft material morphogenesis, but new technologies are needed to change metal shape while retaining its advantageous mechanical properties.

We present an approach for achieving large changes in the mechanical properties of stiff and high‐strength cellular metals through controlled topological changes activated by electrochemically connecting and disconnecting lattice struts. The stiffness of a steel lattice unit cell reversibly switches between 1.1 MPa and 2.6 GPa with minimal change in relative density, while arrays of lattices allow access to stiffness states between the unit cell limits. Compared to other stiffness‐changing materials, our materials achieve the highest stiffness state and change with the least power per volume due to the efficiency of electrochemical systems. A topology optimization technique that solves for two extreme topologies with minimal change in part volume enables lattices with switchable positive and negative Poisson's ratios. Electrochemical plating provides electrically controllable and reversible topology changes at room temperature while maintaining the advantageous properties of metals in both topological states. Electrolyte and lattice material selection maintain the lattice geometry during 200 plating and etching cycles. Finally, we demonstrate an in situ phase control of a vibrating mass through stiffness modulation in encapsulated lattices.

## 2 Results and Discussion

### 2.1 Programmable Stiffness Through Changes in Topology

**Figure 1:**
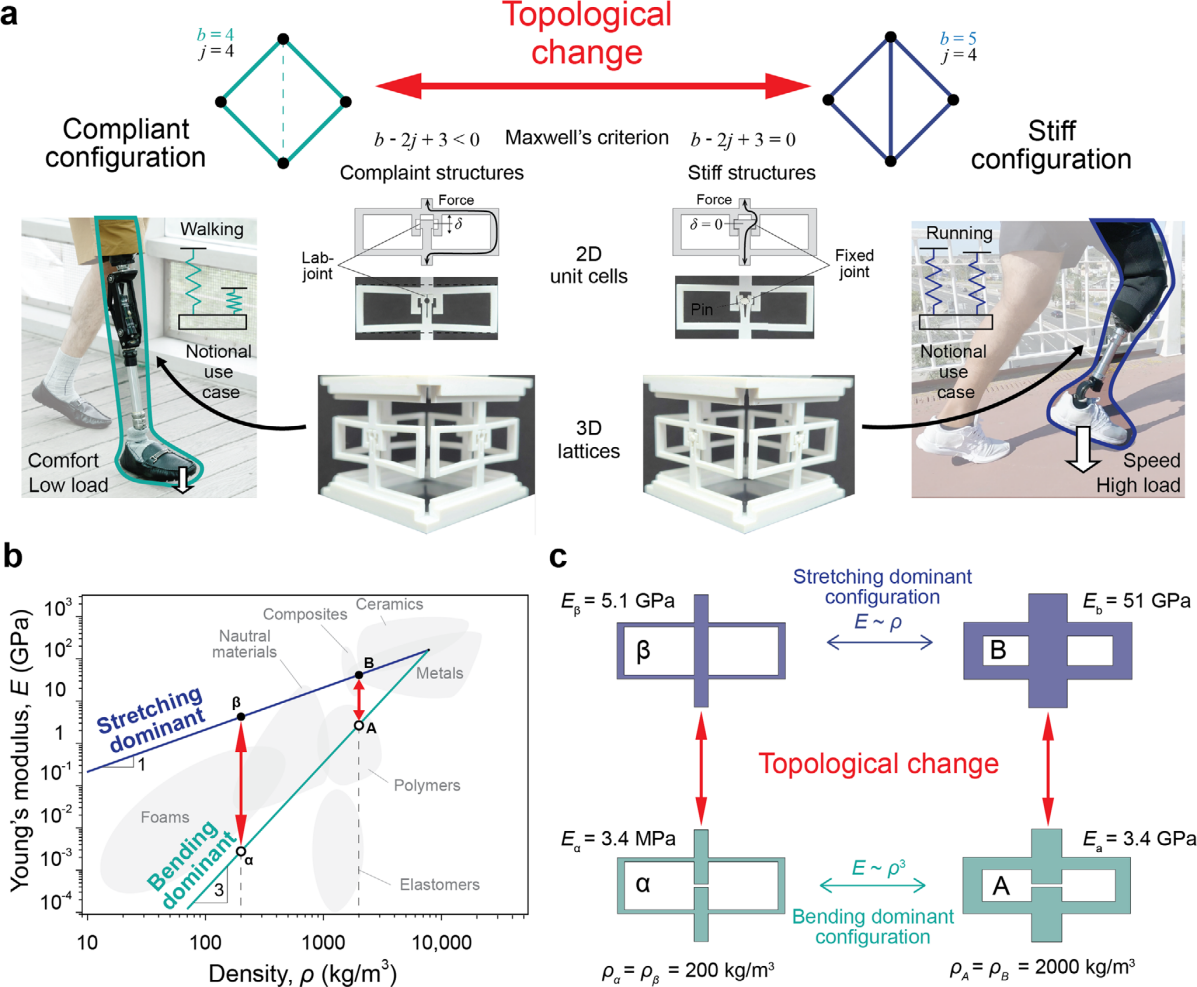
Controlling mechanical behavior through topological changes. a) A notional example of modulating the stiffness of a prosthetic to optimize comfort and speed when switching between running and walking. The programmable stiffness is implemented through topological changes. In Maxwell's stability criterion equation(b − 2*j* + 3), *b* represents the number of struts (lines), and 𝑗 represents the number of joints (dots). By adding a strut crossing the center in a compliant (bending‐dominant) configuration, the equation b − 2*j* + 3 = 0 is satisfied, transforming it into a stiff (stretching‐dominant) configuration. The bending‐stretching transition is demonstrated through a 3D printed plastic rectangular unit cell with a lab‐joint in the central strut. The concept can be expanded to 3D lattices by assembling or combining unit cells. b) A plot of the dependence of Young's modulus on density for a 2D rectangular unit cell, following a scaling law. Points α, β, A, and B correspond to the Young's modulus for a stretching and bending dominated lattice at 200 and 2000 kgm^−^^3^. The shaded areas show the properties of common engineering materials (from Granta Design). c) Schematics of the geometry, density, and Young's modulus of lattices that correspond to points α, β, A, and B. Movements on the blue and teal lines indicate changes in relative density (strut thickening). In contrast, vertical red arrows represent changes through topology modification with minimal differences in relative density.

The notional use of a prosthetic limb in **Figure** **1**a demonstrates how programmable stiffness can enable new device functionalities. The dynamics of prosthetics can be modeled with a single spring whose stiffness is the primary parameter affecting key mobility metrics.^[^ ^31^, ^32^ ^]^ While walking, the spring constant should be low to smoothly adjust the foot displacement to the surrounding environment and improve comfort by reducing sudden impact to the body (left – Figure 1a). On the other hand, prostheses used while running should have a high spring constant to rapidly convert large body forces into forward momentum (right – Figure 1a). Current prosthetics require users to have multiple prosthetics with uniquely tuned dynamics to accommodate various activities like walking and running. In addition, the prosthetic stiffness needs to be proportional to the body's weight,^[^ ^31^ ^]^ so a new prosthetic is required if the user's weight changes. Materials that could change stiffness at the magnitude of human body forces and with the robustness of metals would transform the design landscape of prosthetics and other similar technologies, by allowing a single device to achieve optimal performance under changing operational goals and allow the same device to grow and adapt with the user.

We use topology changes to realize programmable stiffness because small changes in topology can significantly affect mechanical properties, which could enable the walking/running transition desired in prosthetic applications (Notional illustrated in Figure 1a). Consider the simple rhombus structure shown in Figure 1a. This structure easily deforms under tension or compression because the struts that connect the nodes bend when the top and bottom nodes are pulled. This topology is classified as bending dominant according to Maxwell's stability criterion,^[^ ^20^, ^22^ ^]^ since *b* − 2*j* + 3 < 0, where b represents the number of struts and 𝑗 represents the number of joints. If a connection is added between both end nodes, however, the structure's Maxwell's stability criterion is satisfied, *b* − 2*j* + 3  =  0, and its deformation is stretching dominant because the new strut resists the tensile force without bending. In this manner, the addition and removal of this single strut significantly changes the deformation mode of the rhombic lattice. Figure 1a (middle) shows this transition implemented in plastic 3D printed rectangular unit cells. The central strut of the unit cell is rigidly connected or disconnected via lab joints. When disconnected, tensile or compressive forces applied to the unit cell are transferred through bending beams in the outer rectangle. After connecting the central strut by inserting a pin, force is primarily transferred through the stiff central strut. This can be expanded to any cellular material in 2D or 3D. Figure 1b shows the advantage of using topology changes to enable metals with programmable stiffness.

Figure 1b shows the advantage of using topology changes to enable metals with programmable stiffness. Although topology‐driven stiffness change is material agnostic, we focus the remaining of our work on metals because of their importance in modern civilization and the lack of technological approaches available for dynamically changing their properties. The teal line shows how lattice stiffness changes with relative density for a bending dominant topology (E ≈ *ρ* ^3^), while the blue line represents a stretching dominant topology (E ≈ *ρ*). For a material that keeps the same topology, significant changes in relative density are required to alter stiffness. Achieving a 1000 times stiffness change in a bending‐dominated lattice (α ↔ A) requires a 10 times change in density. For the stretching‐dominated lattice, the same change in density only increases stiffness by 10 times (β ↔ B). In contrast, topological changes efficiently enable large stiffness variations with minimal change to relative density. For example, transitioning between α and β requires a topology change but can achieve a 1000 times stiffness change at the same relative density, which is the same as transitioning from the stiffness of elastomers (MPa) to soft metals (GPa). A challenge is to identify topologies and mechanisms that allow lattices to easily transition between bending and stretching‐dominated. We achieve this by making a stretching‐dominated lattice that is one strut removed from a bending‐dominated lattice. We then cut this critical strut (Figure 1c), which is usually oriented parallel to the loading direction, and then reversibly weld and unweld the cut strut to transition the lattice between stiffness states.

### 2.2 Electrochemical Plating and Etching for Switching Topology

**Figure 2:**
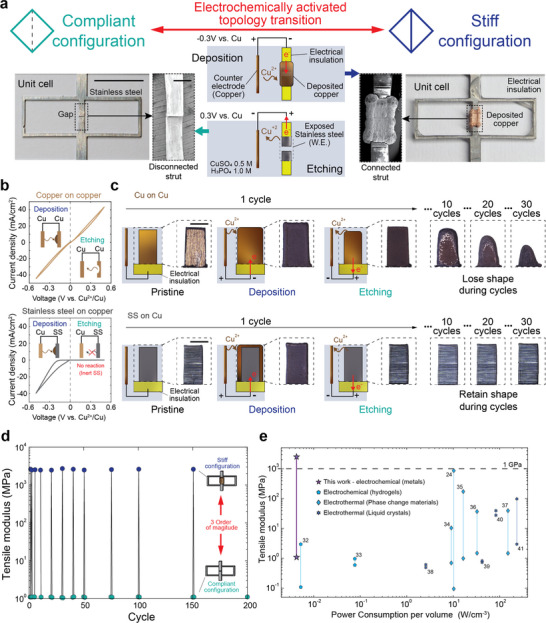
Reversible topology transitions via electrochemically controlled morphogenesis with low‐power density. a) A topology change (red arrow) through electrochemical activation. The left shows a compliant unit cell, which can turn stiff after electrochemical deposition (top center). Except for the connection area, the metal surface is insulated and used as a working electrode facing the counter electrode (copper) in the electrolyte. The stiff configuration can be restored to the original compliant state through copper etching (bottom center) by applying a reverse voltage. b) Cyclic voltammetry (CV) on copper and steel lattice materials. The steel is inert to oxidation. c) Confirmation of shape retention during the cycling process. Protrusions representing half of the connection area are repeatedly plated/etched with 5 mAh capacity. Stainless steel protrusions retain their shape while copper gradually loses its shape. Scale bar, 1 mm. d) Tensile test results of a lattice unit cell in the compliant and stiff configuration during cycling. In each cycle, the lattice is electrochemically transitioned from a compliant state to a stiff state and then returned to the original state. The mechanical properties were measured in select cycles (1–5,10, 20, 30, 40, 50, 100, 150, and 200). e) A plot comparing programmable stiffness technologies with electrical control. The high and low points show the stiffness range, and the power density is the input power normalized by volume of active material, based on ref. [11].

We used electrochemical copper plating and etching to connect and disconnect lattice struts because it is the only metal welding technique that can connect metal parts at room temperature, with electrical control and no external intervention (in‐operando), and maintain the original metal strut strength and electrical conductivity.^[^ ^15^, ^33^ ^]^ **Figure** **2**a shows a 2D rectangular lattice unit cell cut from a 304 Stainless Steel metal sheet with an 8 kW laser cutter (Mazak OPTIPLEX). The center strut of the rectangular lattice is cut in half with a fine gap (≈100 µm) to allow this part to be connected and disconnected. After insulating the structure except for the cut region, including a 1.5 mm length of the adjacent area, we use the metal structure as a working electrode and place a counter electrode made of the metal intended for plating into the electrolyte. Applying a negative potential causes copper ions in the electrolyte to reduce and accumulate at the cut, mechanically bridging the gap and inducing a topology change in the lattice. Applying the opposite potential causes the metal to oxidize into ions, disconnecting the strut. Electrical power is only needed to transition between topologies, not to maintain a topology. Based on this approach, we can construct a system that goes back and forth between two topologies with distinct mechanical properties through a synthetic morphogenesis controlled by the potential difference between two electrodes.

We found that the lattice structural material should be electrochemically inert within the oxidation potential range of the connecting metal to allow the lattice to maintain its original shape after repeated plating and etching cycles. Figure 2b shows a voltammogram of copper plating on copper and steel. The steel has no reaction under the copper etching potential (<0.5 V), which enables precise etching of only the deposited copper. In contrast, the copper lattice loses its initial shape under periodic cycles as portions of the base metal get slowly etched (Figure 2c – top) while the steel maintains its shape (Figure 2c – bottom).

Electrochemical plating and etching enable lattices with reversible mechanical properties. Figure 2d shows stiffness measurements of the lattice unit cell in Figure 2a after 200 cycles of electrochemically welding, measured under tensile testing (Instron 68SC‐2). Compressive deformation can be achieved in addition to tensile deformation through the overlapping of connecting struts (Figure S4, Supporting Information). In each cycle, we apply 3000 pulses (with a 33% duty cycle of 1‐s on, 2‐s off) to establish the connection, followed by continuous plating until the total deposition amount is achieved (total input charge of 20 mAh, including the pulse plating). The etching process uses a constant potential of 0.3 V with the same amount of input charge, 20 mAh. The tensile modulus was 1.1 MPa in the compliant state and 2.6 GPa in the stiff state, demonstrating a 2500 times stiffness change with only 0.017 MPa and 0.074 GPa standard deviation during 200 cycles. The connection (deposition) process takes ≈3 h, while disconnection (etching) requires ≈2.5 h. There is an opportunity to further reduce the transition time to 1 h or less through electrolyte optimization (Section S6, Supporting Information). Response times on the order of hours are sufficient for many applications, such as structures that adapt to weather changes or prosthetics used intermittently (≈9 h a day). The advantage of the high modulus also makes our technology well‐suited for applications involving metal structures requiring modulus changes (Figure S7, Supporting Information). Figure 2e compares this topology‐driven stiffness change to other electrically‐driven stiffness modulation technologies. The topology‐driven approach has some standout advantages: achieving the highest maximum stiffness while being comparable to the largest ranges of stiffness change. As the electrochemically‐driven topology change only required 0.3 V of applied potential (see Figure S6, Supporting Information), it required the lowest power density of any of the prior studies. Although some of these prior techniques used electrochemical changes to modulate stiffness, the maximum stiffness was limited by the intrinsically low stiffness of soft materials used (hydrogels, pentagon symbols in Figure 2e).^[^ ^34^, ^35^ ^]^ Several phase change (diamond symbols in Figure 2e ^[^ ^30^, ^36^, ^37^, ^38^, ^39^ ^]^ and hexagram symbols in Figure 2e)^[^ ^39^, ^40^, ^41^, ^42^, ^43^ ^]^ materials have been explored for programmable stiffness. Liquid metal^[^ ^30^ ^]^ achieved a high maximum stiffness and a wide range; however, all of these materials required a large power density because of their thermal input. In contrast, the intrinsically low voltage of the electrochemical oxidation‐reduction reactions and the minimal material needed for topology changes enabled the low‐power density in this work.

### 2.3 Lattice Design for Programmable Stiffness

**Figure 3:**
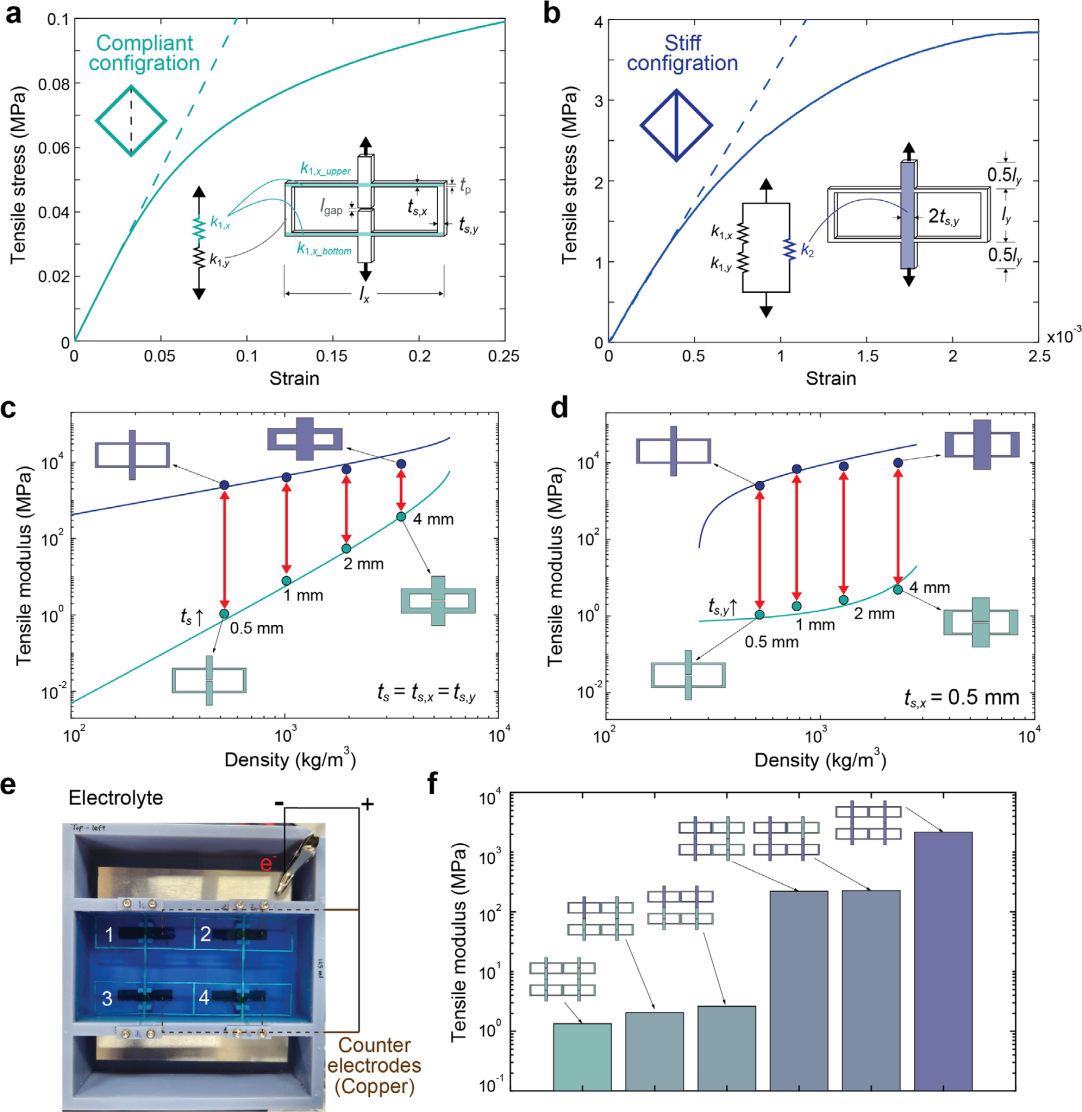
Tensile properties of the unit cell and lattice. a) Tensile test measurements of a unit cell with a compliant topology (dotted line indicates elastic regime), the dimensions of the unit cell with schematics represented as a series of springs. b) Tensile test measurements of a unit cell with a stiff topology. c) Experimental results and theoretical prediction of the unit cell tensile modulus vs density as the width of the *x*‐direction and *y*‐direction struts, *t_s_*_,_ *_x_* and *t_s_*_,_ *_y_* are increased uniformly from 0.5 mm to 1, 2, and 4 mm. d) Experimental results and theoretical prediction of the unit cell tensile modulus vs density when only *t_s_*_,_ *_y_* is increased from 0.5 to 1, 2, and 4 mm while *t_s_*_,_ *_x_* is fixed at 0.5 mm. **e**) A 2×2 lattice with independently addressable unit cells. The lattice is the working electrode, and the counter electrodes are installed at the bottom of the electrolyte container below each unit cell central strut to selectively electroplate each connecting area. f) Stepwise stiffness increase of the lattice between the minimum and maximum limit of the unit cell. The topology state of individual cells is indicated by teal (compliant) and blue (stiff) colors.

We establish a model to predict geometric effects on the programmable stiffness change and then demonstrate that lattices can achieve multiple stiffness states between the upper and lower limits of the unit cell. The spring models in **Figure** **3**a,b represent the rectangular unit cell stiffness. In the compliant topology, the unit cell behavior is dominated by the bending horizontal struts (teal shaded, *k*_1,_ *_x_*), while the two vertical struts connecting these horizontal struts (*k*_1,_ *_y_*) are barely deformed. In the stiff topology, the central vertical strut (blue shaded, *k*_2_) is connected and resists axial deformation, so that the unit cell shows ≈100 times less strain even under ≈10 times higher stress (Figure 3a,b).

We measured the elastic modulus from the linear elastic region of the unit cell tensile tests (Dash lines in Figure 3a,b) and show how unit cell dimensions affect this property. Assuming ideal springs and 1‐D displacement, the elastic modulus, *E*, depends on the unit cell width and height (*l_x_*, *l_y_*) and the thickness of each strut (*t*_s,_ *_x_*, *t*_s,_ *_y_*) by

(1) $$E_{c}=16E\left(\frac{l_{y}}{l_{x}}\right){\left(\frac{t_{s,x}}{l_{x}}\right)}^{3}$$

(2) $$E_{s}=2E\left(\frac{t_{s,y}}{l_{x}}\right)$$

where the subscripts c and s represent the compliant and stiff topology (See supplementary information for derivation). Analyzing the case where the thickness of the strut is the same (*t*_s_ = *t*_s,_ *_x_* = *t*_s,_ *_y_*), each modulus increases in proportion to the cube and the first power of *t*_s_, respectively, and the ratio of the two moduli, *E*_s_/*E*_c_, is inversely proportional to the square of *t*_s_,

(3) $$\frac{E_{s}}{E_{c}}=\frac{l_{x}^{3}}{8l_{y}}\frac{1}{t_{s}^{2}}∼\frac{1}{t_{s}^{2}}$$

The stiffness ratio is ≈3000 when *l_x_ =* 45 mm*, l_y_* = 15 mm, and *t*_s_ = 0.5 mm. Increasing the length of the cell, *l_x_*, further increases the ratio (*E_s_*/*E_c_* ≈*l_x_* ^3^). Figure 3c shows that even though the maximum stiffness increases, the ratio rapidly decreases as the thickness of the strut uniformly increases. To increase both the stiffness ratio and maximum stiffness, we fix the horizontal thickness, *t*_s,_ *_x_*, which governs the compliant behavior, so that *E_s_*/*E_c_* ≈*t_s,y_* / *t_s,x_^3^* ≈*t_s,y_*. Figure 3d shows the resulting unit cell stiffness. The maximum stiffness increases with density, and the stiffness ratio remains high. The unit cell geometry can be tuned to realize a range of stiffness states while maintaining a high stiffness ratio.

Connecting multiple unit cells into a lattice makes it possible to access many stiffness states between the limits of the unit cell. To explore stepwise stiffness change, we combined the previously used unit cells into a 2 by 2 lattice. Figure 3e shows the lattice in an electrical bath that allows control over each unit cell stiffness using four separated counter electrodes. Figure 3f shows the stepwise change in stiffness as the number of cells is changed from the compliant topology (teal) to the stiff topology (blue). When all cells are in the compliant or stiff topology, the lattice stiffness matches the unit cell, but we can access (2n)! / 2(n!) additional stiffness states in n by n lattices (SeeSection S7, Supporting Information for derivation).

### 2.4 Demonstration of In Situ Topological Transitions

**Figure 4:**
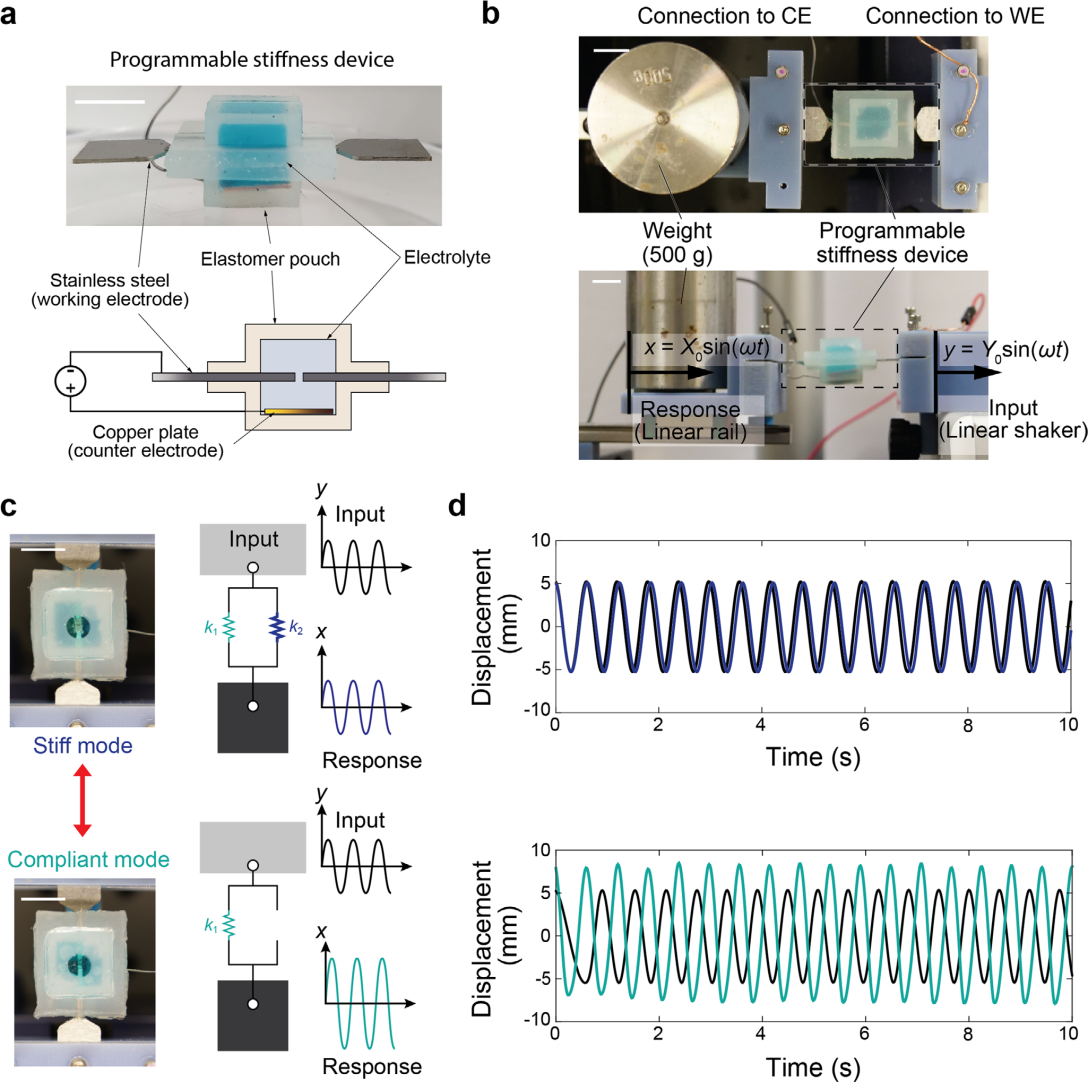
Programmable stiffness material for in situ control of vibrational responses. a) A schematic of the programmable stiffness material. An elastomer pouch holds a small amount of electrolyte (1 mL), and applying a voltage between the embedded counter electrode and lattice allows in situ control of mechanical properties. Scale bar, 10 mm. b) An experimental setup to test the vibration response of the programmable stiffness material. One side of the device is connected to a linear slide rail loaded with a 500 g weight, while the other side is connected to a linear shaker, which gives a sinusoidal displacement input. c) Images of encapsulated lattice in the stiff (top) and compliant (bottom) topologies. Spring models with expected outputs for given inputs. Transparent elastomer (PDMS) is inserted to observe the connection states. Scale bar, 10 mm. d) Displacement graphs over time, showing input (black lines) and output (colored lines). In the compliant state, the low stiffness of the spring causes the natural frequency to approach the input frequency (*ω* = 10 rad s^−1^), resulting in an amplified oscillation amplitude.

To achieve in situ control of a dynamic system, we encapsulated our electrochemical system and miniaturized it to fit within the unit cell area. The encapsulated lattice enables the portable material with programmable stiffness envisioned in Figure 1. **Figure** **4**a shows the programmable stiffness material made of a stainless‐steel lattice encapsulated with an elastomer pouch and filled with copper electrolyte. Similar to animal tissue, the material is composed of stiff and soft components connected by intercellular fluids. A copper counter electrode connected by a tap wire is placed inside the pouch at the bottom.

Machines or tools, such as prosthetics, are often harmonically excited by external forces. We attached our encapsulated lattice to a mass, *m*, and subjected it to a periodic displacement, *y*  = *Y*_0_ sin (ω*t*), to test the lattice's ability to adjust stiffness while responding to external stimuli (Figure 4b). The mass displacement, *x*  = *X*_0_ sin (ω*t*), depends on the lattice displacement and can be predicted by the amplitude ratio *X*_0_/*Y*_0_ =  1/[1 − (ω/ω_n_)^2^], where the stiffness affects the natural frequency by $\omega_{n}=\sqrt{k/m}\;$. The system mass, excitation amplitude, and excitation frequency were fixed at 0.5 kg, 5 mm, and 10 rad s−1. The high and low stiffness of the encapsulated lattice were *k*_stiff_ = 2755 N mm^−1^ and *k*_soft_ = 0.156 N mm^−1^. We tested and extracted the input and mass response as shown in Figure 4c,d with image analysis and fitted each to a sinusoidal wave using nonlinear least‐squares(See Section S8, Supporting Information for detailed fitting data). The amplitude only differs by 2.47% in the stiff topology since the measured input frequency *ω* = 10.6 rad s^−1^ is much smaller than the natural frequency of the system *ω*_n_ = 2,347 rad s^−1^ (ω ≪ ω_n_). When the lattice is in the soft topology, the system's stiffness is determined by the elastomer pouch stiffness, and the frequency ratio becomes close to unity $\omega ∼\;\;\omega_{n}=17.7\;\mathrm{rad}/s$, leading to an amplified response (Figure 4c,d – bottom). The amplified displacement ratio 1.61, which is in good agreement with the theoretical value, *X*_0_/*Y*_0_ = 1/[1− (*ω*/*ω*_n_)^2^] = 1.58. This demonstration shows a direct application of the programmable stiffness material for vibration control of mechanical systems, which are often represented by an excited spring and mass.

### 2.5 Extending Topological Changes to Auxetic and Non‐Auxetic Behavior

**Figure 5:**
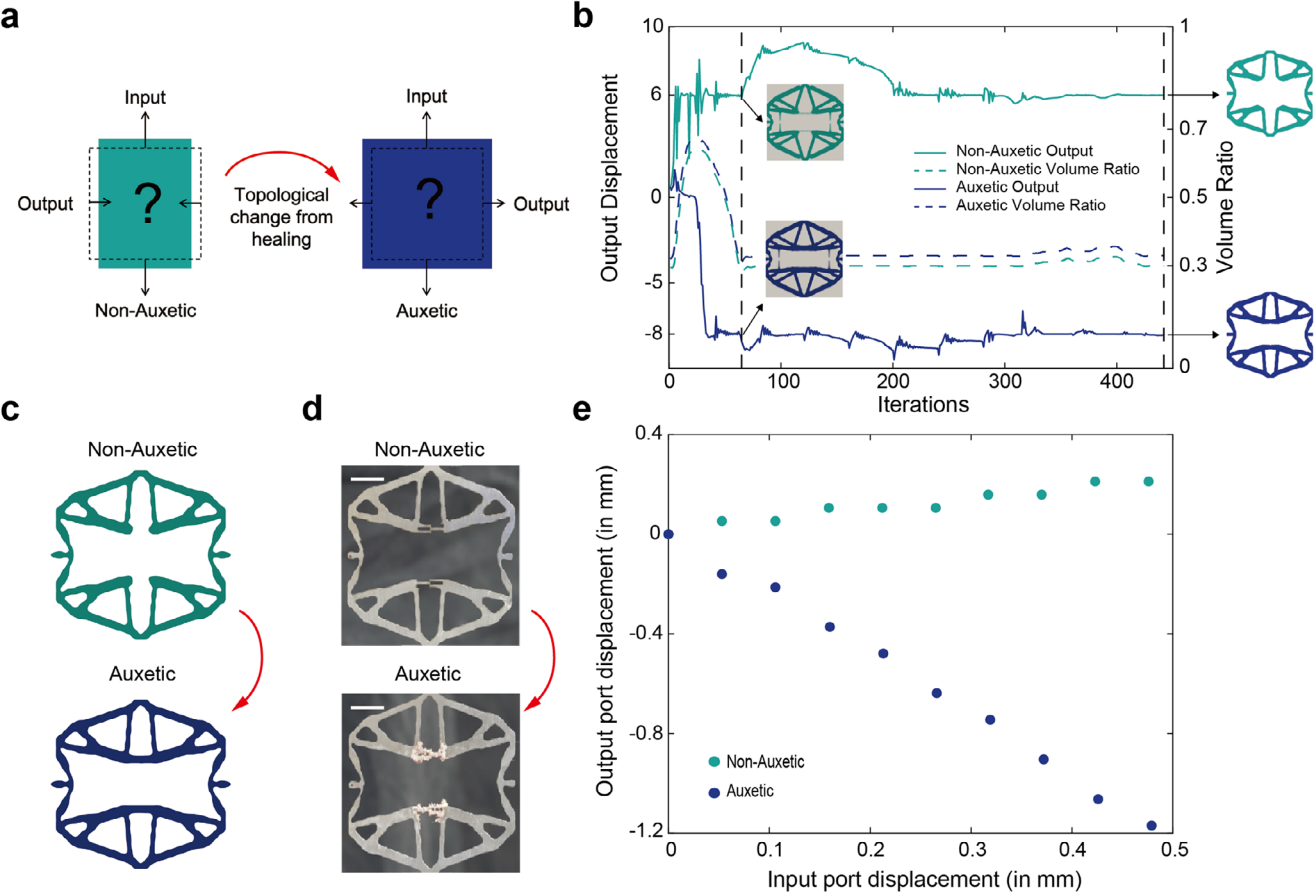
Topology optimization to enable electrochemically‐driven transition between auxetic and non‐auxetic states. a) Conceptual schematic of a mechanical metamaterial which enables switching between non‐auxetic and auxetic behavior using electrochemically activated topology change. b) Evolution of designs during optimization **c**) Optimized geometries to maximize the algebraic difference between the outputs (horizontal displacement) of the non‐auxetic/auxetic behaviors. d) Actual stainless‐steel samples fabricated by laser cutting. Scale bar 10 mm. e) Tensile test results showing the output (horizontal displacement) with non‐auxetic and auxetic behaviors.

Electrochemically activated topology changes can be used to program many lattice properties, including the ability to transition materials between auxetic and non‐auxetic states. We used topology optimization to design a structure that can switch between an auxetic and non‐auxetic state by connecting and disconnecting major struts. **Figure** **5** shows the design process and experimental results.

The design domain is a square modeled using a mesh of 160 × 160 bilinear finite elements. The design variable for each element is local volume fraction, which can vary between zero (indicating the element is part of a hole) and one (indicating material is present in the element). Although volume fraction is a continuous variable to allow use of gradient‐based optimizers, we use a standard penalization approach^[^ ^44^ ^]^ for driving designs to clear 0–1 (void‐solid) layouts and the projection method for controlling the minimum length scale of designed features, thereby ensuring manufacturability of designs.^[^ ^45^, ^46^ ^]^ To capture topological changes enabled by electrochemical welding, we use two sets of topological variables: standard projection variables associated with the base (non‐auxetic) topology and new projection variables representing the electrochemically deposited material. The auxetic structure design is then generated by combining both variables. In the interest of experimentally demonstrating this idea on a single unit cell, this design challenge is cast as a compliant mechanism topology optimization problem where we maximize the algebraic difference of the output port displacements (located at the arrows in Figure 5a) between the two configurations under an input load. To ensure meaningful designs, we require a minimum stiffness in both the auxetic and non‐auxetic states, assume a spring stiffness at the output ports, and place volume constraints on the total volume of the structure and on the volume of the electrochemical connections used. As this problem desires maximal motion and thus hinge‐like connections,^[^ ^47^ ^]^ ^49^ we use the robust topology optimization methodology to prevent any one‐node (one‐point) connections.^[^ ^48^, ^49^ ^]^

Figure 5b shows the design evolution during optimization and the convergence of various properties when solving the problem with the gradient‐based Method of Moving Asymptotes optimizer.^[^ ^50^, ^51^ ^]^ It can be seen from the plot that the design begins to take shape around iteration 65 and eventually converges (iteration 442) to a clear design where volume constraints are satisfied and the non‐auxetic and auxetic outputs satisfy the required displacement magnitudes. Figure 5c depicts the final optimized designs.

The primary topological mechanism for stiffness change is the two horizontal struts near the middle of the structure, which are absent in the non‐auxetic structure and present in the auxetic structure. To facilitate electrochemical welding, a channel design was used for these horizontal struts, as shown in Figure 5d. In the non‐auxetic state, this channel acts like a cut beam, allowing the structure to contract laterally with only minimal resistance resulting from frictional contact. This channel is then welded shut to create a rigid strut, allowing the structure to achieve the auxetic state. Although this design modification and assumed boundary conditions cause a quantitative deviation from the theoretical predictions, the qualitative behavior of the phase change is consistent in experiments (Figure 5e).

## 3 Conclusion

This work demonstrates the advantage of programming cellular material properties by changing the lattice topology via electrochemically controlled morphogenesis. Topology changes from bending to stretching‐dominated lattices allowed stainless steel to achieve a programmable stiffness between 1.1 MPa and 2.6 GPa. Spring models of the unit cells predicted the stiffness limits, while unit cell arrays allow access to intermediate stiffness states. Topology optimization designed lattices that transition between auxetic and non‐auxetic states, opening the possibility of programming many desired mechanical properties through topology change. Electrochemical welding allowed electrical control over the topology change at room temperature while also connecting struts without sacrificing their high strength and stiffness. Mechanical cycling measurements showed that it is possible to achieve chemically and mechanically reversible topology changes with low energy input through judicious selection of electrolyte and lattice material. The combination of topology changes and electrochemical welding work together to enable the lowest power density materials with electroprogrammable stiffness change while also enabling portability, as demonstrated with in situ stiffness control of vibrational responses. Through this research, we endowed metals with the morphogenetic properties of living organisms, which were previously intractable.

## 4 Experimental Section

### Fabrication of Unit Cells and Lattices

The unit cells and lattices are prepared by laser‐cutting from a 304 Stainless Steel metal sheet(0.61 mm‐thick) with an 8 kW laser cuter (Mazak OPTIPLEX). The detailed dimensions are shown in the drawing in Figure S3 (Supporting Information). Metal structures are coated with an insulation material (Parylene C or nail polisher), excluding the cut area, to have metal ions reduced only at the connection area, enabling efficient transition.

### Electrochemical Process and Mechanical Test

A lab‐made aqueous copper sulfate solution was used as an electrolyte for all the experiments. Copper(II) sulfate pentahydrate and phosphoric acid are added in ultrapure deionized water at concentrations of 0.5  and 1.0 molL^−1^, respectively. A copper plate (McMaster‐Carr, Multipurpose 110 Copper) with a thickness of 0.53 mm and a width of 25 mm was used as the counter electrode, ensuring that the area of the counter electrode was larger compared to the plated area of the working electrode(≈10 mm^2^). The electroplating setup ensures suppressing the loss of electrolytes due to evaporation(Figure S5, Supporting Information). 3000 cycles of pulsed plating(one cycle includes plating at −0.3 V vs Cu for 1 s followed by 4s at open circuit voltage) is applied before applying potentiostatic plating at −0.3 V vs Cu until reaching 20 mAh. For the etching process, 0.3 V vs Cu was applied until the current became less than 0.1 mA. Cyclic voltammetry was performed using (Biologic) at 10 mVs^−1^. Stainless steel and copper plate(1 cm × 1 cm area; the rest of the surface was insulated) were tested as working electrodes, while a Cu plate as both the reference electrode and counter electrode. The protrusion samples(Figure 2c) were made by cutting a 0.53 mm‐thick copper plate and a 0.61 mm‐thick stainless steel plate. The protrusions extend 2 mm outward with a 1 mm‐wide tip from a 10 mm‐wide base. Tensile testing of unit cells and lattices was performed in a mechanical testing machine(Instron 68SC‐2) equipped with a 50 N(for unit cells) and 2 kN load cell(for lattices) and wedge‐action grips at a speed of 0.05 mm min^−1^.

### Fabrication of a Programmable Stiffness Device

A pair of half‐dogbones, which has a neck cross‐section of 1 mm × 0.61 mm, was designed to be connected by electrochemical welding and used as a stiff structural part of the programmable stiffness device. An elastomer(Eco‐flex 00–10, Smooth‐On) was used as a compliant part while encapsulating the electrolyte and counter electrode. The elastomer pouch was fabricated using by aluminum mold. Half of the parts of the elastomer pouch were made of an aluminum mold separately and joined to have an internal void with a size of 1  × 1  × 1 cm and a 2 mm‐thick wall. Copper was used as the counter electrode(1  × 1 cm area). Stainless wire provides an electrical connection to an external power source. All joints and seals were made using silicone rubber adhesive(Sil‐poxy, Smooth‐On). After assembly, a lab‐made electrolyte was injected using a syringe, and then the injection hole was sealed. For devices with a viewport, a hole was punched on one side of the pouch using a 5 mm punch, and PDMS elastomer was cured to create a transparent section.

### Real‐Time Vibration Response Test

A linear shaker (SCI‐L330‐Pro, SCILOGEX) was used for linear harmonic displacement. A linear rail was used to enable horizontal movement of the weight, and the mounts were made using a 3D printer (Object30, Stratasys). The grips at both ends of the device's stiff part are connected to the linear shaker and the weight. The displacements of linear shake(input) and weight(output) are recorded by a camera and characterized by image processing in MATLAB.

### Topology Optimization

Details on the topology optimization formulation are described in the supplementary information section S9 (Supporting Information).

## Conflict of Interest

The authors declare no conflict of interest.

## Supporting information



Supporting Information

## Data Availability

The data that support the findings of this study are available from the corresponding author upon reasonable request.
