# Supplementary material for: Morphogenetic Metals through Topology‐Driven Stiffness Changes and Electrochemical Activation
Source: Small. 2025 Oct 31;21(47):e10823. doi: 10.1002/smll.202510823 (PMC12658919; doi:10.1002/smll.202510823)
Supplement: Supplementary file 1 — Supporting Information [file SMLL-21-e10823-s001.pdf]

NANO · MICRO
small

Supporting Information

for *Small*, DOI 10.1002/smll.202510823

Morphogenetic Metals through Topology-Driven Stiffness Changes and Electrochemical Activation

*Jungtaek Kim, Yash Agrawal, Zakaria Hsain, James K. Guest and James H. Pikul**

Supporting Information

Morphogenetic metals through topology-driven stiffness changes and electrochemical activation

Jungtaek Kim , Yash Agrawal, Zakaria Hsain, James K. Guest & James H. Pikul.*

Jungtaek Kim, James H. Pikul

Department of Mechanical Engineering, University of Wisconsin-Madison, Madison, WI, USA

E-mail: jpikul@wisc.edu

Yash Agrawal, James K. Guest

Department of Civil and Systems Engineering, Johns Hopkins University, Baltimore, MD, USA

Zakaria Hsain

US Department of Energy, Hydrogen and Fuel Cell Technologies Office, Washington, DC, USA

Supplementary Figs. 1–7, Sections 1–8.

1. Scaling model for the elastically tensile deformation
   1.1 - 3D open-cell foam
   1.2 - 2D rectangular unit cell
2. Geometry of unit cell
3. Unit cell design enabling compressive behavior
4. Detailed description of mechanical cycle test
   4.1 - Preparation of sample
   4.2 - Experimental setup for electrochemical reaction
   4.3 - Mechanical cycle test
5. Comparison of the range of stiffness changes according to applied voltage
6. Comparison of stiffness change according to time required for operation
7. Stepwise stiffness of n × n lattices
8. Data analysis for base excitation test
9. Topology optimization formulation
   9.1 Design domain
   9.2 Design parametrization
   9.3 Maximum regularization
   9.4 Discreteness parameter
   9.5 Optimization Problem Statement
   9.6 Sensitivity analysis

1. Scaling model for the elastically tensile deformation.

1.1 3D open-cell foam

The scaling law between the relative density of lattice materials and the relative modulus in lattice materials is well explained in Gibson & Ashby (1997).[1]

In the case of idealized open-cell foam structures shown as Figure S1, when a compressive force $F(\sim \sigma L^2)$ is applied to a cubic cell of size $L \times L \times L$, the edges of the cell deflect due to bending, leading to the displacement, $\delta \sim FL^3/(E_s I)$. Here, E_s is the modulus of the solid material, and when the cross-section of the strut has a cross-sectional area, $t \times t$, the second moment of the area will be $I = t^4/12$. Since the strain, ε is $2\delta/L$, and considering the modulus of foam $E=\sigma\varepsilon$, it can be shown that $E/E_s \sim t^4/L^4$. Given that the relative density $\rho/\rho_s \sim t^2/L^2$, the relative modulus is proportional to the square of the relative density, $E/E_s \sim (\rho/\rho_s)^2$.

Using a similar approach as described in Gibson and Ashby's book, *Cellular Solids: Structure and Properties* (Chapter 5)[1] or in the Ashby (2006),[2] the deformation behavior of a 2D rectangular unit cell under tensile force was modeled according to its bending and stretching mechanism to derive the relative modulus as a function of dimensions.

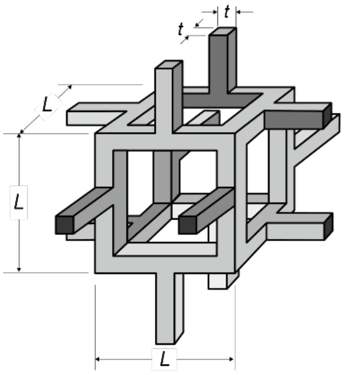

Figure S1. Idealized an 3D open-cell foam as cubic structures with the edge length, *L*, and the edge thickness, *t*.

1.2 2D rectangular unit cell

When a rectangular 2D unit cell with dimensions shown in Figure S2 is subjected to a tensile force *F*, the stress σ can be expressed as $\sigma = F/(l_x t_p)$, where l_x is the horizontal length and t_p is the thickness of the unit cell.

In the compliant unit cell (Figure S2A), both the horizontal and vertical struts will be deformed. However, the most compliant horizontal element undergoes bending so that the largest portion of the displacement is almost equal to the deflections caused by the bending of the horizontal (green) struts. The upper and lower horizontal elements have the same displacement, given by: $\delta_{1,u} = \delta_{1,b} = \frac{FL_{bend}^3}{192EI}$ where L_{bend} is the length of element undergoing bending (here equal to l_x), E is the Young's modulus of the strut material, and I is the moment of inertia of the cross-section of the strut. Thus, the total displacement is

$$\delta = \delta_{1,u} + \delta_{1,b} = \frac{Fl_x^3}{96EI}.$$

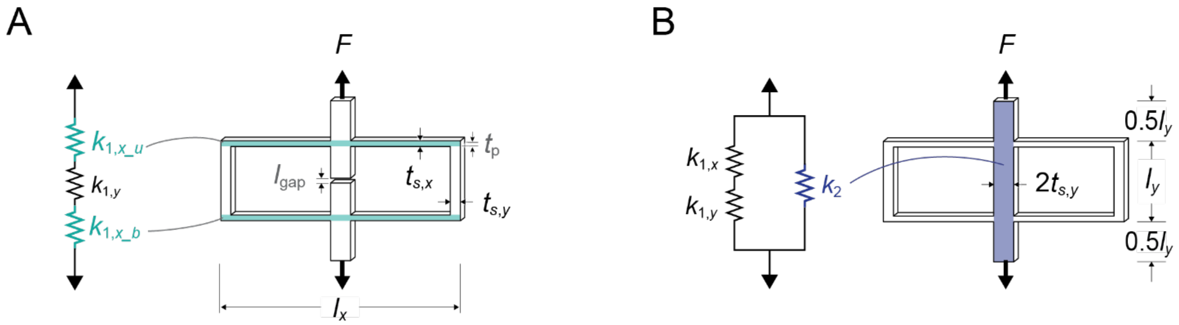

Figure S2. The dimensions of the unit cell at compliant and stiff state with schematics represented as a series of springs.

Since strain is defined as the displacement divided by the original length L in the direction of the tensile force, it is given by

$$\epsilon = \frac{\delta}{L} = \frac{\delta}{2l_y} = \frac{Fl_x^3}{192EIl_y}.$$

Now the modulus of the unit cell can be expressed as follows,

$$E_{\text{compliant}} = \frac{\sigma}{\epsilon} = 16E\left(\frac{l_y}{l_x}\right)\left(\frac{t_{s,x}}{l_x}\right)^3.$$

Since the ratio of the area occupied by the actual material to the total area of the unit is equal to the relative density, the relative density can be expressed as

$$\frac{\rho^*}{\rho} = \frac{2l_x t_{s,x} + 6l_y t_{s,y} - 8t_{s,x}t_{s,y} - 2t_{s,y}l_{\text{gap}}}{\epsilon}.$$

If the widths of the horizontal and vertical struts are the same, $t_{s,x} = t_{s,y} = t_s$. While ignoring the higher-order terms, the relative density is simply proportional to the width of strut, t_s, as seen by

$$\frac{\rho^*}{\rho} = \frac{2l_x t_s + 6l_y t_s - 8t_{s,x}t_s}{\epsilon} = \left(\frac{3}{l_x} + \frac{1}{l_y}\right)t_s \sim t_s.$$

In conclusion, the modulus of a compliant 2D unit cell is proportional to the cube of the relative density.

On the other hand, in case of the stiff state (Figure S2B), the central vertical strut (blue) will bear all the tensile force. The modulus of the unit cell can then be expressed as

$$E_{\text{stiff}} = \frac{2Et_{s,y}}{l_x}.$$

The relative density remains the same as in the compliant case, but the area of the gaps is excluded,

$$\frac{\rho^*}{\rho} = \frac{2l_x t_{s,x} + 6l_y t_{s,y} - 8t_{s,x}t_{s,y}}{\epsilon}.$$

If the widths of the horizontal and vertical struts are the same, $t_{s,x} = t_{s,y} = t_s$, the modulus and relative density can be expressed as

$$E_{\text{stiff}} = \frac{2Et_s}{l_x} \text{ and}$$

$$\frac{\rho^*}{\rho} = \frac{2l_x t_s + 6l_y t_s - 8t_{s,x}t_s}{\epsilon} = \left(\frac{3}{l_x} + \frac{1}{l_y}\right)t_s \sim t_s.$$

The modulus of a 2D unit cell in a stiff state is proportional to the relative density.

2. Experimental dimensions of unit cells

For the ease of tensile testing and electroplating in a bath, we added grips which are fixed at the top and bottom of unit cell with a size of 45 mm × 5 mm. For experimental validation of our mechanical model, we used two set of unit cells varying one dimension.

- Uniform strut width (A): In this set, the width of all struts increased uniformly.
- Varying vertical strut width (B): the width of the horizontal struts was fixed at $t_{s,x}$ = 0.5 mm to maintain a compliant modulus point, while only the width of the vertical struts w as increased. This setup was designed to observe both the increase in maximum stiffnes s and the range of stiffness change.

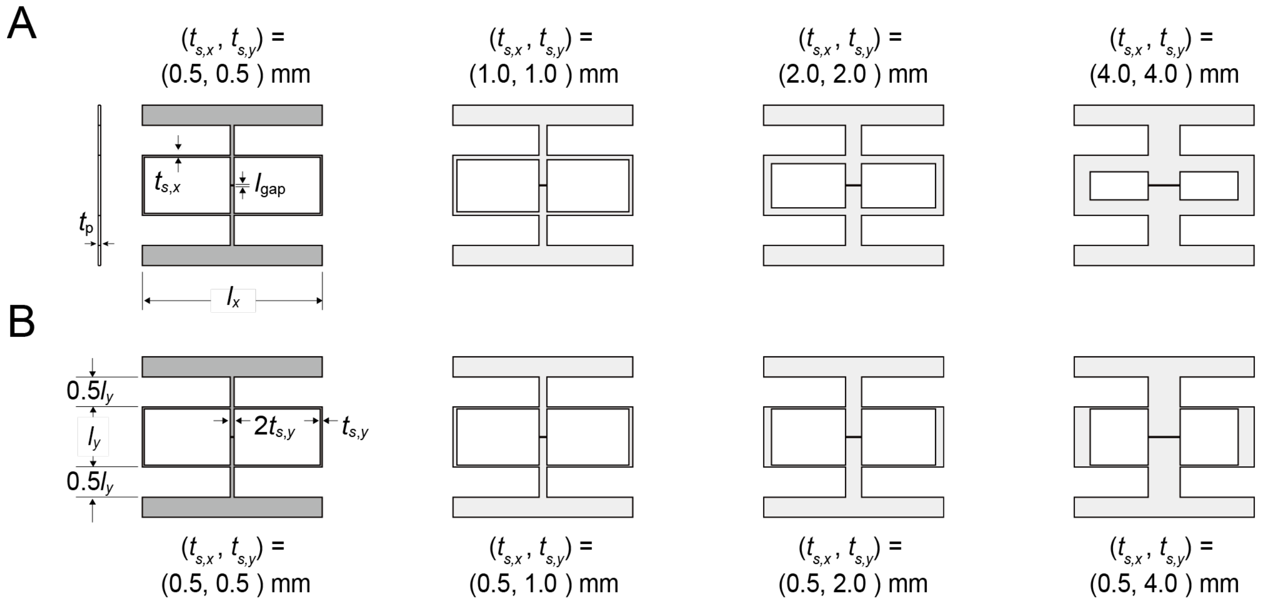

Figure S3. Drawings of all unit cells for experiments

3. Unit cell design enabling compressive behavior

In the current design focused on tensile testing, when plotting the strain-stress curve for the compliant (disconnected) and stiff (connected) states, we observe that in the compliant state, compression occurs normally until the gap size is closed. Once the two boundaries come into contact, the structure exhibits stiff behavior.

To utilize for both tensile and compression behaviors, the overlapping design can be proposed. By applying a structure with an overlap, as shown in the figure, compression can occur in the same manner as in a tensile environment. This allows for a transition to the stiff state, ultimately producing a strain-stress curve similar to that observed in the existing design.

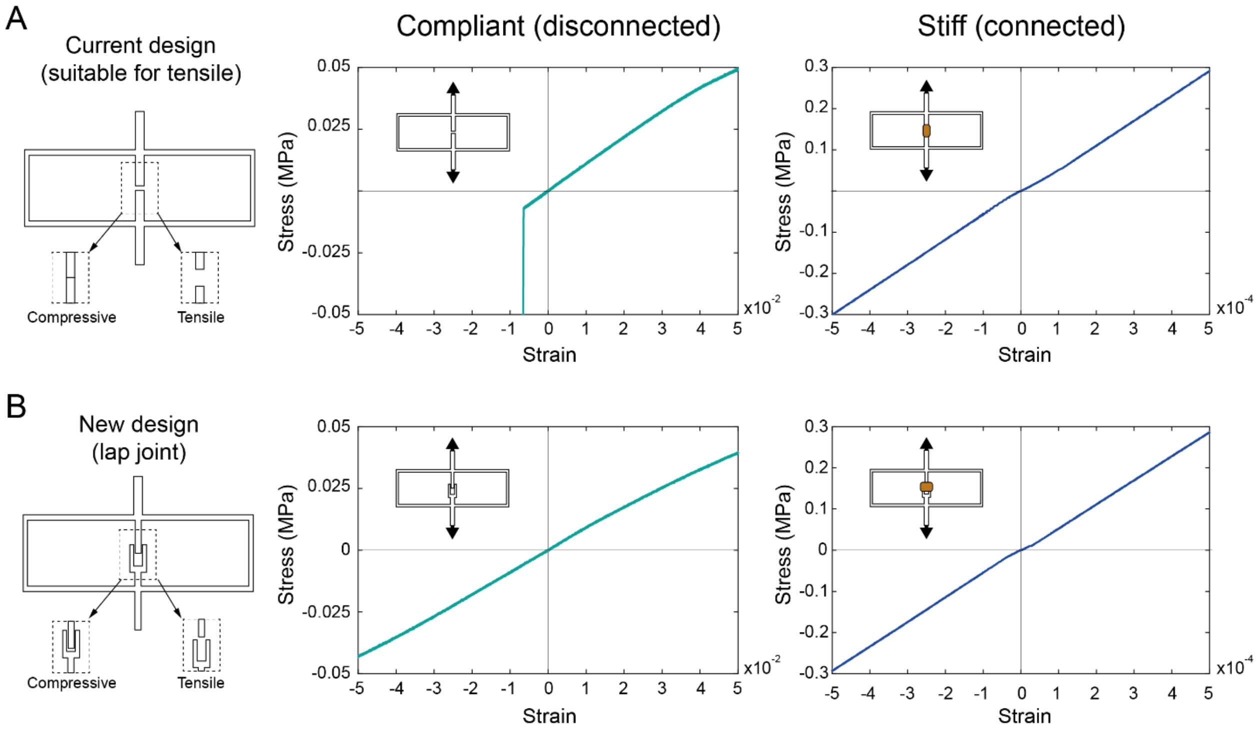

Figure S4. Compressive-Tensile Behavior Based on Unit Cell Design. (A) Unit cell for tensile behavior (used in the main text): In the compliant state, if compression exceeds the gap distance, contact occurs between the structures. (B) Proposed overlap design for compressive behavior: enable proper compression-tensile behavior in both compliant and stiff states.

4. Detailed description of electrochemical-mechanical cycle test

4.1 Sample preparation

Each sample was cleaned and polished to remove contaminants and burrs resulting from laser cutting (Figure S5A). An insulating coating was applied, except for a 3 mm-length area in the connecting area (Figure S5B, C). For the grips, since the coating might deform during mechanical tests, they were insulated with polyimide tape (Kapton®), which was removed before mechanical testing (Figure S5C).

4.2 Experimental setup for electrochemical reactions

The prepared unit cell samples were fixed in a jig as shown in the photos and then immersed in a glass bath (Figure S5D, E). The counter electrode was secured in a glass jar, as depicted in the photos. In this assembled bath (Figure S5E), the sample was used as the working electrode and connected to a potentiostat along with the counter electrode. A potential of -0.3V was applied for welding, and 0.3V for etching while a magnetic stirrer at the bottom of the bath was set to 350 RPM for uniform ion distribution (Figure S5F).

4.3 Mechanical cycle test

To verify mechanical repeatability, we conducted tensile tests on the samples after each electrochemical cycle. The samples were removed from the bath for testing, allowing us to observe the mechanical properties at different stages of the process.

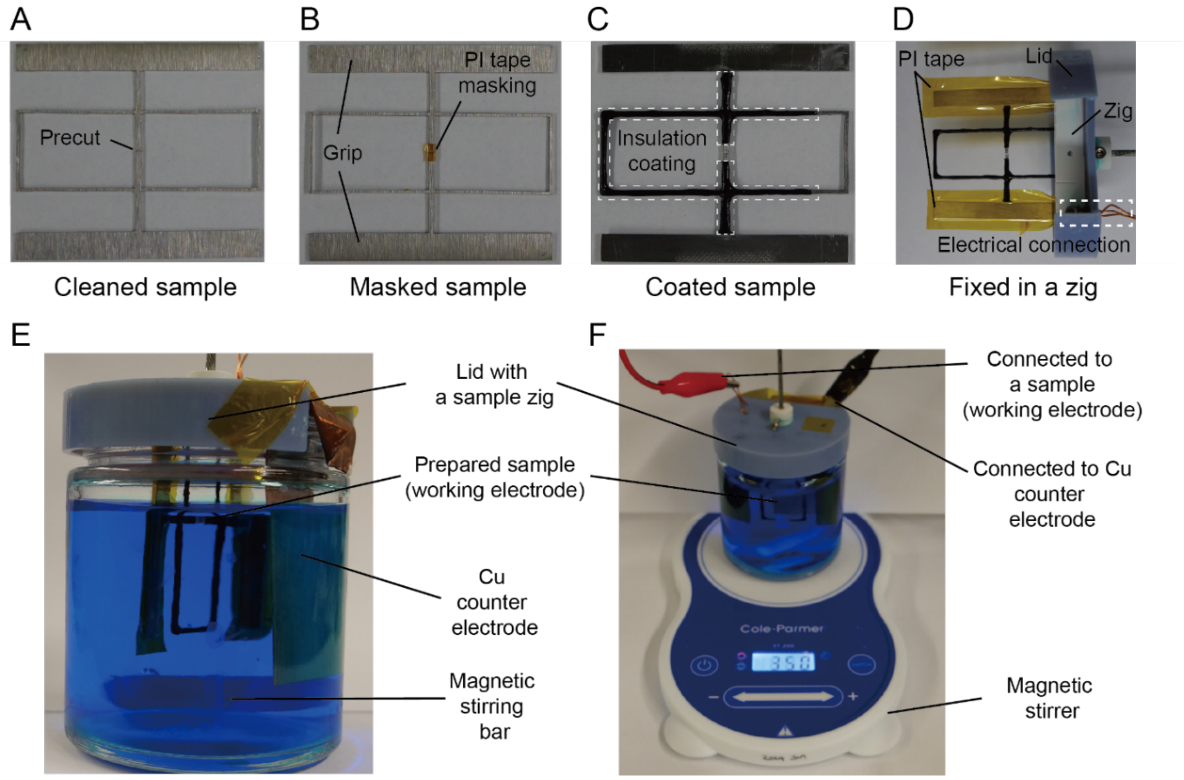

Figure S5. Experimental procedure for electrochemical welding. (A) A cleaned sample after polishing and washing. (B) Masked sample with PI tape strip before applying insulation coating. (C) A sample coated with insulation polymer (nail polish). (D) A sample fixed in a jig before the electroplating process. Grips are masked with PI tape, which can be easily removed before mechanical tests. (E) Electrolytic bath assembled with a lid and a glass jar. (F) Experimental setup for the potentiostat and magnetic stirrer.

5. Comparison of the range of stiffness changes according to applied voltage

Previous research on stiffness control using electrical energy can be categorized into two main groups based on electrical inputs (Figure S6).;

Electrostatic methods (red group)[3-14]: These methods typically utilize Coulombic electroadhesion and generally require high voltages, ranging from 100 to 10,000 V. This requires additional systems for small devices, which is a disadvantage.

Phase change (blue group)[15-27]: This approach involves converting electrical energy into thermal energy (Joule heating) inducing phase changes in materials[15-23] or electrochemical reaction.[24-27] These method requires relatively low voltage (~10 V) but the joule heating demands high power(Figure 2E). In contrast, the electrochemical method (blue pentagon)[24-27] operates at very low voltages because the reduction potential of metal ions is typically around ~1 V, and only a low overpotential (~100 mV) is needed when using the same metal redox couple as the counter electrode. However, previous studies often used hydrogels, which inherently have low stiffness.

Our work (purple star symbol) applies electrochemically activated topological change, achieving not only extremely low voltage operation but also stiffness changes in the range of 10^3 with modulus change in metal structures. This advancement provides significant improvements in stiffness modulation with very low voltage requirements.

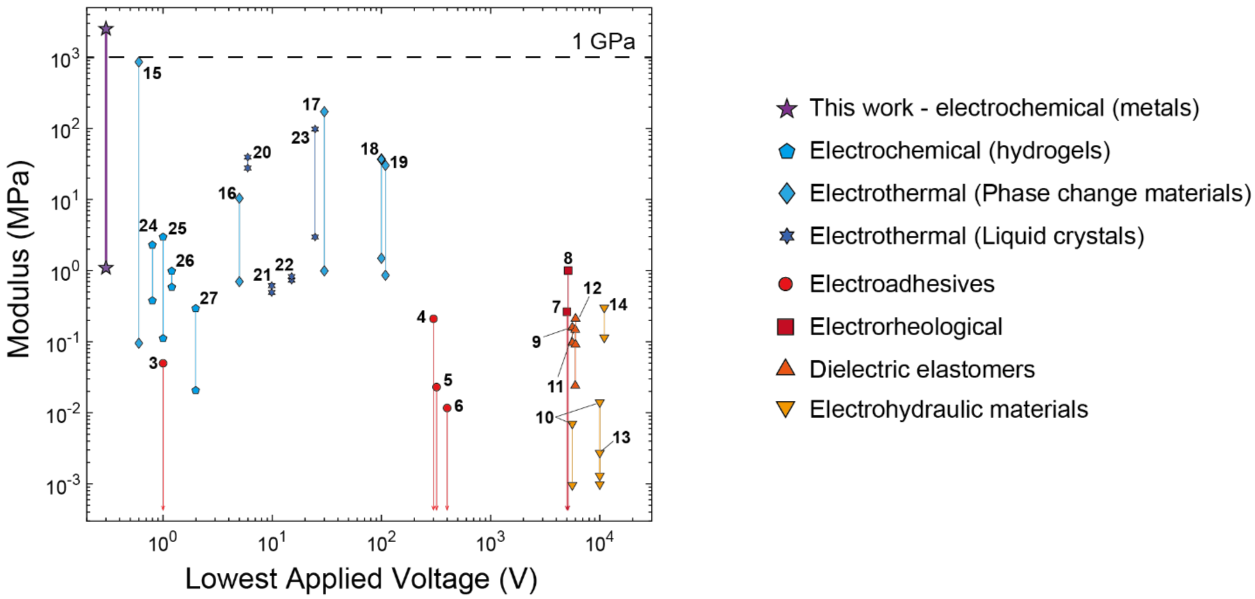

Figure S6. A plot showing the range of stiffness changes according to the lowest applied voltage

6. Comparison of stiffness change according to time required for operation

As shown in Figure S6, which compares various stiffness modulation techniques across different applied voltages (Section 5), we have also compared the time required for operation for each method(Figure S7). The results show a trade-off between response time and modulus range. The red-marked data points represent electrostatic methods, which exhibit extremely fast response times (<1s). However, these methods are limited in their achievable modulus, typically remaining below 1 MPa. In contrast, the blue-marked data points correspond to electrically-activated approaches, which allow for modulus modulation above 1 MPa. However, this comes at the cost of a relatively slower operational speed.

This work shows ~3 hours for operation - the connection(deposition) process takes approximately 3 hours, while disconnection(etching) requires about 2.5 hours. We found that the connection process can be shortened to approximately 1 hour using a commercial plating electrolyte, while etching in a 1 M phosphoric acid solution can be reduced to 1 hour as well. This shows that response time can be further improved through electrolyte optimization.

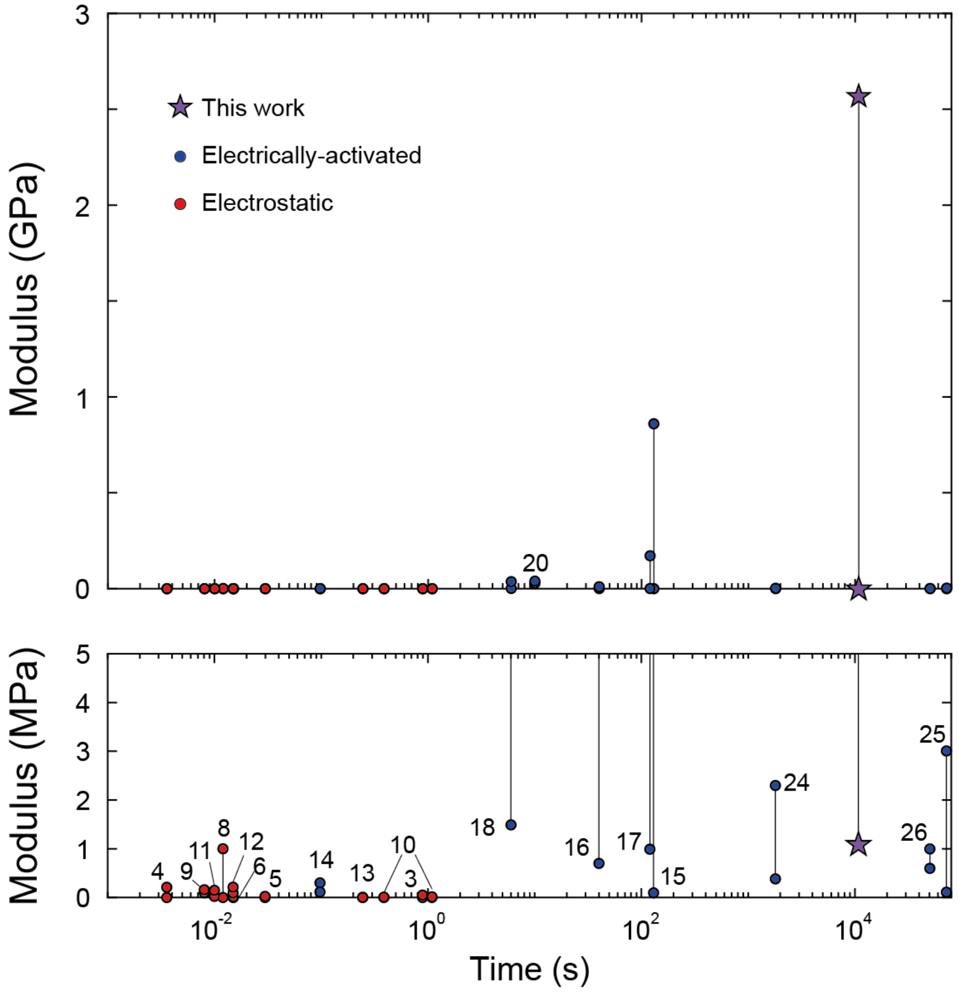

Figure S7. A plot showing the range of stiffness changes according to the time required for activation. For simplification, the red series using the electrostatic methods in the Figure S5 was grouped into a single legend(red circles), and the electrically activated methods was also grouped into a single legend(blue circles).

7. Stepwise stiffness of n × n lattices

In the $n \times n$ lattices (Figure S8A), consider each row as springs connected in parallel, with each row being serially connected to the rows (Figure S8B). We assume only vertical displacement is possible, while rotation is restricted (though actual experiments show some rotation).

In the i^{th}row, the stiffness of the ith lattice, k_i^i, can have a value of either k_c (compliant) or k_s (stiff), like a binary system. The total stiffness K_i of the ith row depends on a_i, the number of lattices activated to stiff state in the ith row, defined by $(n\text{-}a_i)\cdot k_c + a_i\cdot k_s$, where $0 \leq a_i \leq n$. The possible values of K_i thus form a set $\{n\cdot k_c, (n\text{-}1)\cdot k_c + k_s, \cdots, k_c + (n\text{-}1)\cdot k_s, n\cdot k_s\}$, with a total of n+1 values.

The overall stiffness of the system is then defined as:

$$1/\sum_{i=1}^{n}\frac{1}{K_i}$$

Given that the selection process involves choosing one of the n + 1 values n times (without regard to order), this is a combination with repetition problem. Using the formula for combinations with repetition:

$C^*(N,R) = (N + R - 1)! / R!(N - 1)!$ where N is the number of items and R is the number of picks. Substituting $N = n + 1$ and $R = n$ gives: $(n + 1 + n - 1)! / n!(n + 1 - 1)! = (2n)!/(n!)^2$

In the experiment with $n = 2$, this theoretically gives 6 steps, as calculated by $4!/(2!)^2 = 6$. However, due to rotation effects, the actual steps may not be perfectly distinct.

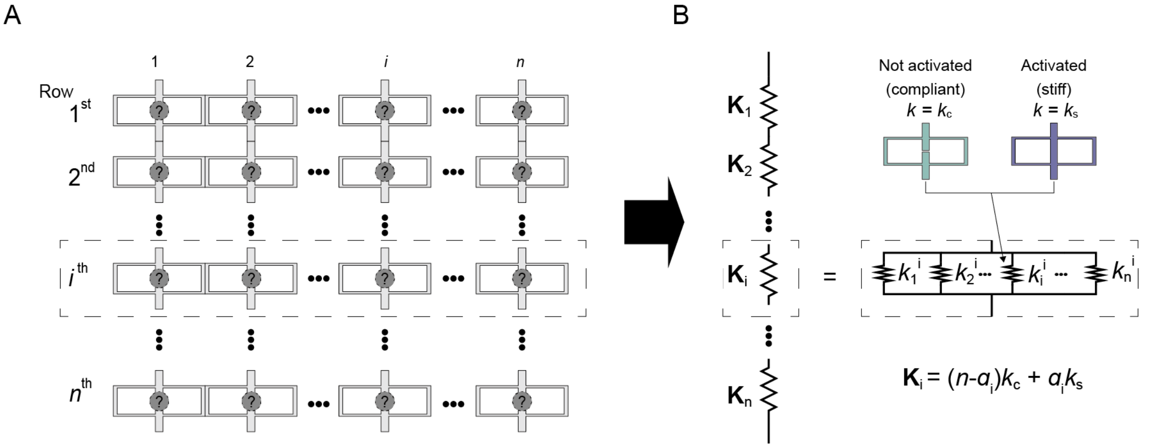

Figure S8. Schematics of deriving the stiffness of $n \times n$ rectangular lattices using a spring model (A) Schematic of $n \times n$ rectangular lattices (B) Lattice replaced with a spring model. It is assumed that there are n parallel connections in a row and that each row is connected in series.

8. Data analysis for base excitation test

The input vibration and mass response shown in Figure 4c,d were fitted with sinusoidal waves, $X=X_0 \sin(\omega t+\phi)$ and $Y=Y_0 \sin(\omega t+\phi)$, using nonlinear least-squares.

Stiff system

Input : $X(t) = 5.26 \sin(10.6t + 1.57)$

Response : $Y(t) = 5.13 \sin(10.6t + 1.11)$

The amplitude only differ by 2.47% since the measured input frequency $\omega = 10.6$ rad/s and the natural frequency of system ω_n=2,347 rad/s, the amplified displacement ratio $X_0/Y_0=1/[1-(\omega/\omega_n)^2] \approx 1$. The phase shift of -26.5° corresponds to a time delay of 44 ms. Considering the video frame rate is 59.92fps, a temporal resolution of 17ms, this shift is within the expected error of imaging and fitting procedures.

Soft system :

Input :$X(t) = 4.83 \sin(10.7t - 2.15)$

Response : $Y(t) = 7.80 \sin(10.7t + 1.40)$

The amplitude is 1.61x lager than the input while the phase also delayed -157° due to damping effect of elastomeric materials of pouch. Using the measured input frequency $\omega = 10.7$ rad/s and the natural frequency of system ω_n=17.7 rad/s, the amplified displacement ratio $X_0/Y_0=1/[1-(\omega/\omega_n)^2] = 1.58$ which is in good agreement with the experimental value of 1.61(=7.80/4.83).

9. Topology optimization formulation

9.1 Design domain

The design domain is a square geometry and represents the unit cell topology. Springs with stiffness k_{in} and k_{out} are attached at the input ports (labelled I and I' in Figure S9) and output ports (labelled O and O' in the Figure S9), respectively. These springs provide resistance to the motion at the ports and represent neighboring bodies at the unit cell connection points, which herein are neighboring unit cells. This approach was implemented as an alternative to inverse homogenization with periodic boundary conditions typically used for architected material design and was chosen because the manufacturing and experimental testing are demonstrated on a single unit cell. Further simulation confirmed auxetic and non-auxetic behavior of optimized designs arranged in finitely periodic patterns.

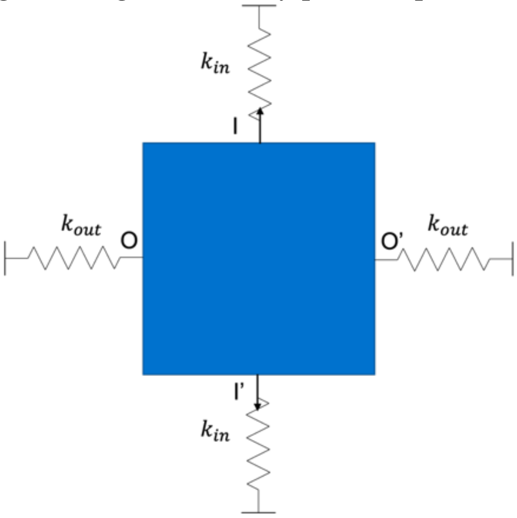

Figure S9. Boundary conditions for the design problem.

9.2 Design parametrization

There are two sets of variables,[31] ϕ_n and ϕ_a, that are used to generate the non-auxetic and auxetic geometries, respectively. Specifically, ϕ_n is the independent design variable field used to generate the volume fraction field ρ_n representing the non-auxetic topology. The field ϕ_a is used to generate the volume fraction field ρ_a that defines the auxetic geometry and equals the summation of ϕ_n and the independent design variable field ϕ_w that represents the welding material design variables. These design variables are located at the nodes of the finite element mesh and each are projected onto element space using the Heaviside Projection Method.[29] We use the robust topology optimization framework[30] to prevent the formation of one-node hinges, which can appear when using low-order finite elements such as the bilinear quadrilateral elements used here. This formulation is modified slightly to achieve dilated and eroded designs by increasing and decreasing the filter radius,[32] respectively.

Following these methods, each of the nodal design variable fields ϕ_n and ϕ_a are passed through a linear filter[33] with different robust filter radii[32] and the resulting filtered fields are then passed through a regularized Heaviside function,[29] denoted as H. This can be written succinctly as:

$$\rho_{j,k}^{i}(\phi_j) = H(\phi_j, r_{min}^{i})$$

where superscript i indicates the blueprint (i=1 or i="b"), eroded (i=2) or dilated (i=3) design from the robust set; subscript j indicates the non-auxetic (j=1 or j="n") or auxetic (j=2 or j="a"); and subscript k is the element number. Resulting designs are analyzed with a finite element solver as needed. Element stiffness matrices associated with each design are defined from the elemental densities using the SIMP[37] material interpolation scheme:

$$\left(K_{j,k}^{i}\right)_{ele} = \frac{1}{1+\rho_{min}}\left(\left(\rho_{j,k}^{i}\right)^{\eta} + \rho_{min}\right)K_{ele}^{0}$$

Here K_{ele}^0 is elemental stiffness matrix with unit volume fraction, η is the SIMP exponent penalty for driving solutions to binary distributions ($\eta > 1$), and ρ_{min} is a small positive number to ensure the finite element global stiffness matrix is positive definite (ρ_{min} =10^{-5}). The displacements at the output port are denoted as scalar variables $S_j^i(\phi_j)$ following the notation described above, with the non-auxetic structure desired to have positive magnitudes of S, and the auxetic structure having negative magnitudes.

9.3 Maximum regularization

Following the robust formulation,[30] the maximum of output displacements of the main, eroded, and dilated designs must be estimated. The mellowmax function is used as a smooth approximation of the max function as follows:

$$S_j^{max}(\phi_j) = \frac{(-1)^j}{\alpha} \log\left[\frac{1}{3}\sum_i e^{\alpha(-1)^j S_j^i}\right]$$

where the regularization parameter is chosen as α=30.

9.4 Discreteness parameter

For the auxetic and non-auxetic designs, a discreteness parameter[34] is computed on the blueprint design (i=1) as:

$$M_j(\phi_j) = \frac{1}{n}\sum_{k=1,nel} 4\rho_{j,k}^i(1-\rho_{j,k}^i)$$

where *nel* is the number of elements. This function equals zero when the elemental volume fractions are binary, and equals one when all elemental volume fractions are equal to one-half (the farthest from binary). To help SIMP drive solutions to binary distributions, the non-auxetic structure discreteness measure is added to the objective function.

9.5 Optimization Problem Statement

The design goal is to maximize the difference in auxetic and non-auxetic displacements using a limited volume of material for the non-auxetic ($V_{n,max}$) and auxetic ($V_{a,max}$) structures. We note that this formulation is chosen to better match the experiments, as we are not performing homogenization on the unit cell design to compute Poisson's ratio. A minimum displacement constraint is applied to the non-auxetic ($S_{n,min}$) structure to ensure some level of stiffness, while a maximum displacement constraint is applied to the non-auxetic ($S_{a,max}$) structure to ensure negative displacement behavior. The resulting optimization problem is expressed as:

$$\begin{aligned} \min_{\phi_n,\phi_w} \quad & S_a^{max}(\phi_n,\phi_w) - S_n^{max}(\phi_n) + cM_n(\phi_n,\phi_w) \\ s.t.: \quad & V(\rho_n^b) \leq V_{n,max} \ ; \ V(\rho_a^b) \leq V_{a,max} \\ & S_{n,min} \leq S_n^b(\phi_n) \ ; \ S_a^b(\phi_n,\phi_w) \leq S_{a,max} \\ & 0 \leq \phi_n \leq 1; \ \ 0 \leq \phi_w \leq 1 \end{aligned}$$

where $V(\rho_n^b)$ and $V(\rho_a^b)$ are the volume of the blueprint non-auxetic and auxetic designs, respectively. The optimization process uses a continuation method on SIMP variable η and projection variable β following typical topology optimization practice.[36]

9.6 Sensitivity analysis

The adjoint method is used to compute sensitivities at every optimization iteration.[28] These sensitivities are defined as the derivative of the objective (or constraint) function with respect to the elemental volume fractions representing the current design. A series of chain rules through the Heaviside function and the linear filter[29] lead us to the sensitivity of these response functions to the nodal variables ϕ_n and ϕ_w, which are ultimately used to guide the design evolution using the gradient-based Method of Moving Asymptotes[35] optimizer.

1 L. J. Gibson & M. F. Ashby. *Cellular Solids: Structure and Properties*. 2 edn, (Cambridge University Press, 1997).
2 M. F. Ashby. The properties of foams and lattices. *Philosophical Transactions of the Royal Society A: Mathematical, Physical and Engineering Sciences* **364**, 15-30 (2006).
3 H. J. Kim *et al.* Low-Voltage Reversible Electroadhesion of Ionoelastomer Junctions. *Adv. Mater.* **32**, 2000600 (2020).
4 R. Hinchet & H. Shea. High Force Density Textile Electrostatic Clutch. **5**, 1900895 (2020).
5 S. B. Diller, S. H. Collins & C. Majidi. The effects of electroadhesive clutch design parameters on performance characteristics. **29**, 3804-3828 (2018).
6 V. Ramachandran, J. Shintake & D. Floreano. All-Fabric Wearable Electroadhesive Clutch. **4**, 1800313 (2019).
7 A. Zatopa, S. Walker & Y. Menguc. Fully Soft 3D-Printed Electroactive Fluidic Valve for Soft Hydraulic Robots. *Soft Robotics* **5**, 258-271 (2018).
8 A. Tonazzini, A. Sadeghi & B. Mazzolai. Electrorheological Valves for Flexible Fluidic Actuators. *Soft Robotics* **3**, 34-41 (2016).
9 W.-B. Li, W.-M. Zhang, H.-X. Zou, Z.-K. Peng & G. Meng. Bioinspired Variable Stiffness Dielectric Elastomer Actuators with Large and Tunable Load Capacity. *Soft Robotics* **6**, 631-643 (2019).
10 V. Cacucciolo *et al.* Stretchable pumps for soft machines. *Nature* **572**, 516-519 (2019).
11 S. Dastoor, M. R. J. I. I. C. o. R. Cutkosky & Automation. Design of dielectric electroactive polymers for a compact and scalable variable stiffness device. 3745-3750 (2012).
12 A. Orita & M. R. Cutkosky. Scalable Electroactive Polymer for Variable Stiffness Suspensions. *IEEE/ASME Transactions on Mechatronics* **21**, 2836-2846 (2016).
13 X. Wang, S. K. Mitchell, E. H. Rumley, P. Rothemund & C. Keplinger. High-Strain Peano-HASEL Actuators. **30**, 1908821 (2020).
14 E. Acome *et al.* Hydraulically amplified self-healing electrostatic actuators with muscle-like performance. **359**, 61-65 (2018).
15 W. Shan, T. Lu & C. Majidi. Soft-matter composites with electrically tunable elastic rigidity. *Smart Mater. Struct.* **22**, 085005 (2013).
16 S. Rich, S.-H. Jang, Y.-L. Park & C. Majidi. Liquid Metal-Conductive Thermoplastic Elastomer Integration for Low-Voltage Stiffness Tuning. *Advanced Materials Technologies* **2**, 1700179 (2017).
17 M. Tatari, A. Mohammadi Nasab, K. T. Turner & W. Shan. Dynamically Tunable Dry

Adhesion via Subsurface Stiffness Modulation. *Advanced Materials Interfaces* **5**, 1800321 (2018).

18 W. Shan, S. Diller, A. Tutcuoglu & C. Majidi. Rigidity-tuning conductive elastomer. *Smart Mater. Struct.* **24**, 065001 (2015).

19 A. M. Nasab, A. Sabzehzar, M. Tatari, C. Majidi & W. Shan. A Soft Gripper with Rigidity Tunable Elastomer Strips as Ligaments. *Soft Robotics* **4**, 411-420 (2017).

20 J. Liu *et al.* Shaping and Locomotion of Soft Robots Using Filament Actuators Made from Liquid Crystal Elastomer–Carbon Nanotube Composites. *Advanced Intelligent Systems* **2**, 1900163 (2020).

21 C. Wang *et al.* Soft Ultrathin Electronics Innervated Adaptive Fully Soft Robots. *Adv. Mater.* **30**, 1706695 (2018).

22 H. Kim *et al.* Intelligently Actuating Liquid Crystal Elastomer-Carbon Nanotube Composites. *Adv. Funct. Mater.* **29**, 1905063 (2019).

23 C. Yuan *et al.* 3D printed reversible shape changing soft actuators assisted by liquid crystal elastomers. *Soft Matter* **13**, 5558-5568 (2017).

24 J. T. Auletta *et al.* Stimuli-Responsive Iron-Cross-Linked Hydrogels That Undergo Redox-Driven Switching between Hard and Soft States. *Macromolecules* **48**, 1736-1747 (2015).

25 R. D. Harris *et al.* Chemical and Electrochemical Manipulation of Mechanical Properties in Stimuli-Responsive Copper-Cross-Linked Hydrogels. *ACS Macro Letters* **2**, 1095-1099 (2013).

26 P. Calvo-Marzal *et al.* Manipulating Mechanical Properties with Electricity: Electroplastic Elastomer Hydrogels. *ACS Macro Letters* **1**, 204-208 (2012).

27 E. Palleau, D. Morales, M. D. Dickey & O. D. Velev. Reversible patterning and actuation of hydrogels by electrically assisted ionoprinting. *Nat. Commun.* **4**, 2257 (2013).

28 O. Sigmund. On the Design of Compliant Mechanisms Using Topology Optimization , Journal of Structural Mechanics **25**(4), 493-524 (1997).

29 J. K. Guest, J. H. Prévost & T. Belytschko. Achieving minimum length scale in topology optimization using nodal design variables and projection functions. *International Journal for Numerical Methods in Engineering* **61**, 238-254 (2004).

30 O. Sigmund. Manufacturing tolerant topology optimization. *Acta Mechanica Sinica* **25**, 227-239 (2009).

31 J. K. Guest. Topology optimization with multiple phase projection. *Computer Methods in Applied Mechanics and Engineering* **199**(1–4), 123–135 (2009).

32 A. T. Gaynor, N. A. Meisel, C. B. Williams and J. K. Guest. Multiple-Material Topology Optimization of Compliant Mechanisms Created Via PolyJet Three-

Dimensional Printing. *ASME. J. Manuf. Sci. Eng.* **136**(6): 061015 (2014).

33 T. E. Bruns, and D. A. Tortorelli. Topology Optimization of Non-Linear Elastic Structures and Compliant Mechanisms. *Comput. Methods Appl. Mech. Eng.* **190**(26–27), 3443–3459 (2001).

34 Sigmund, O. Morphology-based black and white filters for topology optimization. *Struct Multidisc Optim*. **33**, 401–424 (2007).

35 K. Svanberg. The method of moving asymptotes—a new method for structural optimization. *International Journal for Numerical Methods in Engineering* **24**, 359-373 (1987).

36 J. K. Guest, A. Asadpoure and S. H. Ha. Eliminating beta-continuation from Heaviside projection and density filter algorithms. *Struct Multidisc Optim* **44**, 443–453 (2011).

37 M. P. Bendsøe. Optimal shape design as a material distribution problem. *Structural Optimization* **1**, 193–202 (1989)
